# Influence of Lycopene Intake on Mental Health: A Systematic Review of Randomized Controlled Trials (RCTs)

**DOI:** 10.3390/nu17111793

**Published:** 2025-05-25

**Authors:** Dominika Głąbska, Dominika Guzek, Anna Jílková, Aleksandra Kołota-Burdzy, Dominika Skolmowska, Lenka Kouřimská

**Affiliations:** 1 Department of Dietetics, Institute of Human Nutrition Sciences, Warsaw University of Life Sciences (SGGW-WULS), 159C Nowoursynowska Street, 02-776 Warsaw, Poland; dominika_glabska@sggw.edu.pl (D.G.); aleksandra_kolota@sggw.edu.pl (A.K.-B.); dominika_skolmowska@sggw.edu.pl (D.S.); 2 Department of Food Market and Consumer Research, Institute of Human Nutrition Sciences, Warsaw University of Life Sciences (SGGW-WULS), 159C Nowoursynowska Street, 02-776 Warsaw, Poland; 3 Department of Microbiology, Nutrition and Dietetics, Faculty of Agrobiology, Food and Natural Resources, Czech University of Life Sciences Prague, 129 Kamycka Street, 165-00 Praha, Czech Republic; jilkovaanna@af.czu.cz (A.J.); kourimska@af.czu.cz (L.K.)

**Keywords:** lycopene, intake, status, diet, tomato, supplement, mental health, mental disorders, depression, quality of life

## Abstract

**Background/Objectives:** The potential influence of lycopene on mental health was indicated in some studies, but it was not summarized within any systematic review so far. The aim of the presented study was to analyze the influence of lycopene on mental health within a systematic review of Randomized Controlled Trials (RCTs). **Methods**: The study was conducted according to the Preferred Reporting Items for Systematic Reviews and Meta-Analyses (PRISMA) guidelines, and it was based on PubMed, Web of Science, Cochrane, and Google Scholar databases, while the RCTs published until February 2025 were included. The systematic review was registered within the database of the International Prospective Register of Systematic Reviews (PROSPERO) (CRD420250650525). The studies were considered where the adult population was studied; intervention was based on oral lycopene intake in any form (lycopene supplement, lycopene-enriched functional food, or regular food product being an important source of lycopene); lycopene intake of a specified dose was applied; any mental health result was studied using a valid psychological measure. After duplicate removal, 642 studies were screened, and finally, six RCTs were included and assessed using the revised Cochrane risk-of-bias tool for randomized trials, while various mental health outcomes were allowed (excluding subjects with intellectual disabilities, eating disorders, and neurological disorders). Each stage of screening, inclusion, reporting, and assessment was conducted independently by two researchers. **Results**: The included studies were conducted in populations of healthy individuals (one study), but mainly in individuals with various diseases: Benign Prostatic Hyperplasia (BPH) (two studies), Burning Mouth Syndrome (BMS) (one study), xerostomia (one study), and infertility (one study). Within the included studies, various lycopene sources were applied, including lycopene supplements, functional foods, and regular food products, as well as various lycopene doses from 1.35 mg to 27.8 mg per day. The included studies assessed quality of life (five studies), depression and anxiety (two studies), stress (two studies), and mood states (one study). In spite of the fact that all six included studies were RCTs, the comparison between the intervention group and placebo group was made in only four studies, and none of them stated the difference between the compared groups. A low risk of bias was concluded for three studies (all of them not confirming the influence of lycopene on mental health), and a high risk of bias was found in three studies (one of them not confirming, and two not conclusive). **Conclusions**: The evidence gathered within the systematic review of RCTs did not confirm any influence of lycopene on mental health. Further RCTs are needed to verify the influence of lycopene provided within supplements, functional foods, and regular food products on various mental health problems in diverse populations.

## 1. Introduction

Lycopene is one of the phytochemicals within the group of carotenoids, characterized by many health benefits and a number of potential applications, mainly due to its antioxidative properties [1]. As described by Vogele [2], it was discovered in 1876 by Millardet [3], but it was unnamed until 1903, when Schunck [4] proposed a name based on the Latin name of the tomato (*Lycopersicum esculentum*), where it was detected for the first time. Tomato has since been confirmed as a major source of lycopene, but other sources include pink and red grapefruit, papaya, and persimmon [5].

Among the most prominent studies indicating the role of lycopene, there are mainly those based on the assessment of cardiovascular risk factors. In a meta-analysis of observational studies by Song et al. [6], a preventive role of lycopene was indicated for cardiovascular diseases, including coronary heart disease and stroke. Similarly, the systematic review and meta-analysis of epidemiological evidence by Cheng et al. [7] indicated that a high intake of lycopene, or its high serum concentration, was associated with a decreased risk of stroke, cardiovascular diseases, and mortality. The systematic review and network meta-analysis by Rattanavipanon et al. [8] indicated the influence of lycopene in decreasing systolic blood pressure, which was stated for both healthy and hypertensive individuals. Moreover, it should be indicated that the systematic review and meta-analysis by Cheng et al. [9] confirmed the positive effect on lycopene for blood lipids, blood pressure, and endothelial function, while it was concluded that the effect is observed not only for the supplement, but also for the increased intake of tomato and tomato products. However, a systematic review and meta-analysis by Tierney et al. [10] confirmed the potential role of lycopene in improving cardiovascular risk factors, but with some conflicting results across studies, independently from the applied dose and the mode of delivery (dietary intake or supplementation).

It should be mentioned that the potential role of lycopene is indicated also for other diseases and medical conditions. The systematic review by Inoue et al. [11] indicated the positive effect of lycopene intake on the fasting blood glucose level. The systematic review and meta-analysis by Chen et al. [12] noted that a higher blood lycopene level and higher lycopene intake were associated with a lower risk of prostate cancer. The meta-analysis by Li and Xu [13] also suggested a potential role of dietary lycopene in the risk of ovarian cancer in postmenopausal women. Similarly, the systematic review and dose-response meta-analysis of prospective cohort studies by Balali et al. [14] confirmed that dietary lycopene intake and blood lycopene level were associated with a lower risk of overall cancers and death due to cancer, while for mortality, the association was also observed for the total tomato intake. Last but not least, the systematic review by López-Valverde et al. [15] indicated that lycopene may be used as an adjunctive treatment for periodontal disease.

Among various potential beneficial health effects of lycopene: anticancer, antioxidative, hypocholesterolemic, cardioprotective, anti-diabetic, and anti-aging [16], there are some potentially related not only to physical health, but also to mental health, which should be taken into consideration [17]. The intake of compounds with antioxidative properties is associated with depression, anxiety, and sleep disorders, and potentially also with other mental health problems, while the potential mechanisms are associated with reduced amount of free radicals, resulting in reduced lipid peroxidation in brain tissue, reduced brain neurons damage, and reduced neurotransmitters imbalance [18]. Similarly, impaired cholesterol metabolism, including hypercholesterolemia [19], as well as blood glucose level, are associated with an increased risk of mental health problems [20]. Moreover, the intake of some compounds with confirmed anti-aging properties, e.g., resveratrol, is indicated as beneficial for mental performance [21], so the influence of other components, such as lycopene, may also be hypothesized.

Taking this into account, within a systematic review on lycopene and its potential beneficial effects, the influence on mental health, including depression, was indicated as the area that should be analyzed, due to the fact that there are some studies indicating its potential role, and that based on the mechanisms of action, the influence of lycopene on mental health may be justified [22]. As no such systematic review has been conducted so far, the aim of the presented study was to analyze the influence of lycopene on mental health within a systematic review of Randomized Controlled Trials (RCTs). Taking into account that randomization reduces bias and RCTs are indicated as a rigorous tool to examine cause–effect relationships between an intervention and outcome [23], only RCTs were chosen as the basis for the systematic review.

## 2. Materials and Methods

### 2.1. The Design and Registration of the Systematic Review

The systematic review was conducted according to the Preferred Reporting Items for Systematic Reviews and Meta-Analyses (PRISMA) guidelines [24], and it was registered in the database of the International Prospective Register of Systematic Reviews (PROSPERO) (CRD420250650525).

Studies published until February 2025 were allowed, and the procedure of literature searching was based on PubMed, Web of Science, Cochrane, and Google Scholar databases. Only RCTs were included to compare the mental health results obtained in the intervention group and the control group, while the changes in mental health results observed within the intervention group were not crucial. The included studies were assessed using the revised Cochrane risk-of-bias tool for randomized trials.

### 2.2. The Searching Strategy and the Assessment of the Eligibility

An electronic search was conducted to gather RCTs regarding the influence of lycopene on mental health. The studies were considered while meeting the following inclusion criteria:

1. Adult population studied;
2. Intervention based on oral lycopene intake in any form (lycopene supplement, functional food if lycopene-enriched, or regular food product if being an important source of lycopene);
3. Lycopene intake of a specified dose applied;
4. Any mental health result studied using a valid psychological measure (subjective and objective measures allowed, including the quality of life if indicated to be associated with mental health);
5. Study published in English;
6. Study published in a peer-reviewed journal.

The following exclusion criteria were applied:

1. Animal model study;
2. Study not defined as RCT;
3. Lycopene intervention not compared with placebo;
4. Intervention including multiple factors combined (if impossible to attribute observed effect to lycopene only or mainly, due to a number of applied factors);
5. Studied population of pregnant or lactating women;
6. Studied population of patients with eating disorders;
7. Studied population of patients with intellectual disabilities;
8. Studied population of patients with neurological disorders.

The population, intervention/exposure, comparator, outcome, and study design (PICOS) criteria for the conducted systematic review are presented in Table 1.

### 2.3. The Procedure of Searching and Data Extraction for the Systematic Review

The detailed electronic search strategy applied for PubMed, Web of Science, Cochrane, and Google Scholar databases separately is presented in Table 2.

**Figure 1:**
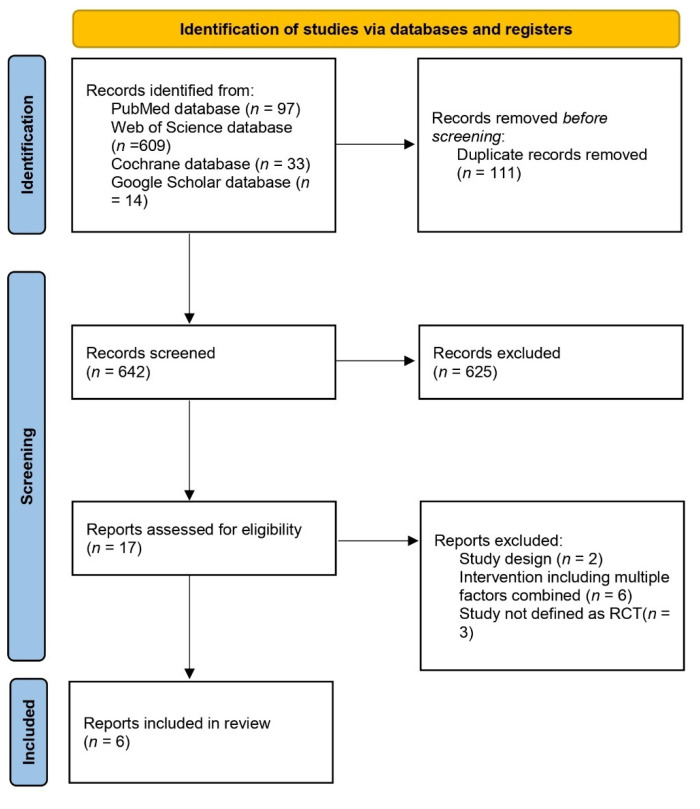
The procedure of identifying, screening, and including studies.

The procedure of identifying, screening, and including studies is presented in Figure 1. The records identified within the searching procedure were manually screened for duplicates, which were removed. The procedure of records screening was made in two stages—based on the titles of articles only, and abstracts of articles (conducted for records initially included based on titles), while it was conducted by two researchers independently, and if any disagreement appeared, the third researcher was invited to participate in making a decision. Afterwards, two researchers independently assessed the eligibility of articles based on the full texts (conducted for records initially included based on abstracts of articles), which was conducted on the basis of previously planned inclusion and exclusion criteria, and if any disagreement appeared, the third researcher was invited to participate in making a decision. The full texts were obtained from the electronic databases and libraries, and for those not available, the corresponding authors were asked for full texts.

The included studies were analyzed to extract the necessary data to describe the study, the studied group, the applied intervention, and the results of the study. The extracted data describing the study were as follows: study design, country and location, brief characteristics of the studied group, time when the study was conducted, and the study duration. The extracted data describing the studied group were as follows: number of participants, number of male participants, number of female participants, age in the intervention group, age in the placebo group, inclusion criteria, and exclusion criteria. The extracted data describing the applied intervention were as follows: type of intervention (supplement, functional food product, or food product), applied intervention, placebo used, characteristics of diet, and lycopene intake (obtained within the intervention). The extracted data describing the results of the study were as follows: mental health outcomes, mental health measures, and observations (results). The necessary data were extracted from the full texts of the studies, but for those not available within the study (or other studies referred to in the full text), the corresponding authors were asked. Two researchers independently extracted the data from the full texts, and if any disagreement appeared, the third researcher was invited to participate in making a decision.

### 2.4. The Procedure of the Assessment of the Quality of Studies and the Risk of Bias

In order to define the quality of the studies, the risk of bias was assessed [25], while the revised Cochrane risk-of-bias tool for randomized trials with the RoB 2 tool (7.0) [26] was chosen, as this tool is commonly used for randomized trials [27]. The risk of bias was assessed according to the recommendations of the Cochrane Review Group [28].

Within the revised Cochrane risk-of-bias tool for randomized trials, five domains were assessed, including: (1) the risk of bias arising from the randomization process, (2) the risk of bias due to deviations from the intended interventions, (3) the risk of bias due to missing outcome data, (4) the risk of bias in measurement of the outcome, and (5) the risk of bias in the selection of the reported result. After the assessment of the listed domains, the overall risk of bias was assessed [29].

The revised Cochrane risk-of-bias tool for randomized trials allows the definition of the risk of bias within each domain as: (1) low risk of bias, (2) some concerns, or (3) high risk of bias, while in order to formulate the final assessment, each domain must be previously assessed, and summarizing of the results for the single domains is the basis of the final score [27].

Two researchers independently assessed the studies, and if any disagreement appeared, the third researcher was invited to participate in making a decision.

## 3. Results

The extracted data to describe the RCTs included in a systematic review are presented in Table 3. Based on the conducted screening, six RCTs were included in a systematic review [30,31,32,33,34,35]. The included studies were conducted in populations of healthy individuals [32], as well as individuals with various diseases: Benign Prostatic Hyperplasia (BPH) [34,35], Burning Mouth Syndrome (BMS) [30], xerostomia [31], and infertility [33]. The study duration was similar in the included studies, and in the majority of them, it was 12 weeks [30,31,32,33,35]. The included studies were conducted in Spain [30,31], Italy [34,35], Japan [32], and Iran [33].

The extracted data to describe the groups studied within the RCTs included in a systematic review are presented in Table 4. The number of participants within the included studies was quite similar, and it ranged from 31 [35] to 100 [32] individuals, so neither very small samples nor large samples were studied within the included studies. In three studies, the sample included both male and female individuals [30,31,32], while in three other studies, due to the characteristics of the studied group (men with infertility, or men with BPH), only male individuals were included [33,34,35]. It was associated with the other inclusion and exclusion criteria (as presented in the Supplementary Table S1). The included individuals (in both intervention groups and placebo groups) were mainly in their 50s [32] or 60s [30,31,34,35], but in one study, they were in their 30s [33].

The extracted data to describe the interventions applied within the RCTs included in a systematic review are presented in Table 5. Within the studies included in a systematic review, various lycopene sources were applied—lycopene supplements [33,34,35], functional foods (studies assumed to be included if lycopene-enriched) [30,31], and regular food products (studies assumed to be included if being an important source of lycopene) [32]. Taking this into account, all the included RCTs presented interventions based on lycopene intake, and the potentially observed effects were attributed to this nutrient. However, the lycopene intake obtained within the intervention differed; it was the lowest in the case of the studied functional food (lycopene-enriched extra virgin olive oil (EVOO)), as the daily supply was approximately 1.35 mg [30,31]. While for the supplements [33,34,35] and the regular food products, the obtained daily supply was 7–20 times higher [32]. The highest daily supply of 22.0–27.8 mg was obtained for regular food product (semidried high-lycopene tomato variety PR-7, compared with lycopene-free tomato) [32], and for supplements, the daily supply of 9.5 mg [34,35] or 25 mg [33] was obtained. If any information about characteristics of diet was presented, it included previous consumption of food products [32,35], avoiding any additional potential sources of lycopene [32,35], and for semidried high-lycopene tomato variety PR-7, not applying any additional thermal treatment that could influence the reduction of lycopene content [20]. Within the studies, the characteristics of diet were assessed in order to confirm that there was no difference between the intervention group and placebo group [32,33].

The extracted data to describe the results of the RCTs included in a systematic review are presented in Table 6. In the majority of the studies included in the systematic review, the quality of life was assessed [30,31,33,34,35] using the tools for the general population: the Short Form Health Survey (SF-36) [30], and the World Health Organization (WHO) Quality of Life Questionnaire (WHOQOL-BREF) [33], or tools for specific populations with diseases or disorders: the Oral Health Impact Profile 14 (OHIP-14) [30,31], and the International Prostate Symptom Score (IPSS) questionnaire [34,35]. In two studies, depression and anxiety were assessed [30,33] using the Hospital Anxiety and Depression (HAD) [30], and the Depression, Anxiety and Stress Scale, a 21-item questionnaire (DASS-21) [33]. Similarly, in two studies, stress was assessed [32,33] using DASS-21 [33], and combined with fatigue, assessed using the Visual Analog Scale (VAS) questionnaire on fatigue and stress [32]. Moreover, in one study, the mood states were assessed using the Profile of Mood States Second Edition (POMS-2) full-length version for adults [32]. In spite of the fact that all six included studies were RCTs, a comparison between the intervention group and placebo group was made only in four studies, and none of them stated the difference between the compared groups [30,31,32,33]. At the same time, for two studies, no comparison between the intervention and control group was made [34,35].

The assessment of the risk of bias for the RCTs included in a systematic review, conducted based on the revised Cochrane risk-of-bias tool for randomized trials, accompanied by a summary of the observations, is presented in Table 7. For the majority of included studies the comparison between the intervention group and placebo group did not confirm the influence of lycopene on mental health [30,31,32,33], while three of them were of a low risk of bias [30,31,32] and one was of a high risk of bias arising from the randomization process [33]. Two studies were stated to be not conclusive, as no comparison between the intervention group and placebo group was made [34,35], while both were of a high risk of bias arising from deviations from the intended interventions and selection of the reported result [34,35], as well as, for one study, also from some concerns regarding the randomization process [34].

## 4. Discussion

The previous studies suggesting the beneficial role of lycopene for mental health were conducted mainly in animal models, while the observed results were stated to be promising mainly for depression models. In the study by Xu et al. [36], it was stated that lycopene alleviates depression-like behavior in chronic social defeat stress-induced mice, as well as it was concluded that lycopene may be a potential antidepressant to be applied within novel antidepressant therapies. Similarly, in the study by Abd Al Haleem et al. [37], in rats presenting clonidine-induced depression-like behavior, it was stated that lycopene and chrysin exerted antidepressant effects. Moreover, in the study by Deore et al. [38], lycopene alleviated Bacillus Calmette-Guerin (BCG)-induced depressive phenotypes in mice.

However, some studies presented some beneficial effects also for mental health outcomes other than depression, including anxiety and Post-Traumatic Stress Disorder (PTSD). In the study by Jain and Gangshettiwar [39], a combination of lycopene, quercetin, and poloxamer 188 alleviated anxiety and depression in 3-nitropropionic acid-induced Huntington’s disease in rats. At the same time, in the study by Li et al. [40], it was observed that lycopene ameliorates PTSD-like behaviors in mice.

Some human observational studies also suggest that the effect of lycopene improves mental health. The study by Niu et al. [41] indicated statistically significant differences in the number of mild and severe depressive symptoms between groups of individuals aged 70 years and over, stratified by a number of consumed tomatoes and tomato products, while the higher number of servings was associated with a lower risk of depressive symptoms, and no relationship was stated for any other kinds of vegetables. Similarly, within the National Health and Nutrition Examination Survey (NHANES) 2007–2016, it was stated that the higher quartile of daily lycopene intake was associated with the reduced depression risk, compared with the lowest quartile, while the most beneficial was the intake not higher than 10 mg (due to a U-shaped association) [42]. It should be emphasized that within the study by Li and Lan [42], it is suggested that a higher dietary lycopene intake may present a protective effect against depression in adult Americans.

Interestingly, similar suggestions of the potentially positive role of lycopene intake for mental health were also formulated within some of the RCTs included in the presented systematic review, even if the comparison between the intervention group and control group did not state any differences. At the same time, within the included RCTs, for the mental health results, either a similar improvement of the mental health outcomes during the study observation period in the intervention group and placebo group [30,33,35] or the positive effect in the intervention group and no effect in the placebo group [30,34] was stated, but in none of the included studies the statistically significant difference between the intervention group and control group was stated [30,31,32,33,34,35]. However, while RCTs are stated to be a gold standard in effectiveness research [23], the conclusion of positive influence should be based on the comparison of the intervention group and control group, not on the assessment of the change from the baseline in the intervention group only [43].

Authors of the included RCTs were trying to find a reason for no difference between the intervention group and control group, and some of them suggested a strong effect of the applied placebo [30]. Such a suggestion is in agreement with the results of a systematic review and meta-analysis of the placebo effect in mental disorders research, by Fernández-López et al. [44], as they observed an influence of the placebo effect, while comparing a placebo with a passive control, in individuals with anxiety and depression.

The other potential explanation for there being no statistically significant differences between the intervention group and control group in the presented systematic review may be the applied doses. As mentioned above, in the NHANES 2007–2016, a U-shaped association was suggested, with the intake not higher than 10 mg per day indicated as the most beneficial [42]. While comparing this level with the doses applied within the RCTs included in the systematic review, it may be suggested that, in a number of them, the applied doses were either too low (approximately 1.35 mg per day) [30,31] or too high (higher than 20 mg per day) [32,33] to be the most beneficial. The studies presenting the influence of a dose potentially the most effective to obtain the improvement of the mental health outcomes (9.5 mg per day), unfortunately, did not present the comparison or the intervention group and control group, but only assessed the significance of changes from the baseline separately for each group [34,35].

The other issue that should be emphasized is the fact that the included studies presented interventions based on various types of products—lycopene supplements [33,34,35], lycopene-enriched functional foods [30,31], and regular food product being an important source of lycopene [32], while all the included RCTs studied effects potentially attributed to lycopene. For lycopene, it should be pointed out that some studies have indicated that the effectiveness of its various sources (foods and supplements) may differ. It was stated in the review by Burton-Freeman and Sesso [45] comparing the effect of tomato intake and lycopene supplementation on cardiovascular risk factors, that only for blood pressure management was the supplementation more effective, while for the other cardiovascular risk endpoints, tomato intake provided more favorable results. Similarly, for antioxidant capacity and exercise-induced lipid peroxidation, the tomato, as a food product high in lycopene, was found to be more beneficial than supplementation, which was attributed to a potential synergistic interaction of lycopene with other bioactive nutrients, while provided within food products [46]. This effect may be especially important for mental health effects, as the potential mechanisms are associated with reduced amount of free radicals, and reduced lipid peroxidation in brain tissue, resulting in reduced brain neuronal damage, and reduced neurotransmitter imbalances [18].

While concluding based on the included RCTs, the low risk of bias in a number of included studies should be emphasized [30,31,32], reflecting a scientific rigor within a well-controlled study, prepared based on adequate assumptions, with unbiased reporting and interpretation of the results.

Taking into account the public health purposes and practical implications of the conducted systematic review, it should be indicated that the lycopene is in general safe, with the acceptable daily intake (ADI) from all food sources established by the European Food Safety Authority (EFSA) panel as 0.5 mg/kg body weight per day [47], as well as with synthetic lycopene, and lycopene extracts generally recognized as safe (GRAS) when added to various food products [48]. Based on various epidemiological studies, the intake of lycopene of 2–20 mg per day is recommended in the prevention and treatment of various diseases [49]. It corresponds with the results mentioned above of the NHANES 2007–2016, suggesting intake below 10 mg per day as the most beneficial [42], but the typical daily intake in the European populations varies from 0.5 mg to 5 mg per day [50]. It indicates that there is room for improvement in order to obtain the health effects of increased lycopene intake, including cardiovascular benefits [6,7,8,9,10], improvement of blood glucose level [11], some cancers [12,13,14], and periodontal disease [15]. The presented systematic review of RCTs does not indicate such a potential beneficial effect for mental health, but no adverse effect was stated. Considering this fact, in spite of the fact that the conducted study did not confirm any influence of lycopene on mental health in a populations of healthy individuals, and individuals with various diseases, increased intake of lycopene should be recommended to obtain multiple potential health benefits while, for mental health effects, further studies are needed.

This systematic review has several limitations that should be taken into account, while the directions for further studies may be indicated. Firstly, taking into account the described problem with an insufficient number of RCTs, which are still needed to verify the influence of lycopene provided within supplements, functional foods, and regular food products on various mental health problems in diverse populations, the need for further studies should be indicated. The studies included presented interventions based on various types of products: lycopene supplements, lycopene-enriched functional foods, and regular food product being an important source of lycopene. However, the influence of lycopene provided within various types of products may vary, so the studies may be incomparable. The studies also presented various mental health outcomes, which were assessed using various tools, which makes the results hardly comparable. At the same time, the studies presented interventions with various doses of lycopene, but if the U-shaped association for lycopene is suggested [40], further studies should consider the effect of the applied doses. Additionally, the studied populations were small and not representative of the general population, so the following studies should be planned to provide stronger conclusions for general public health purposes. Moreover, although all included studies were RCTs, in some cases, there was no clear presentation of the comparison between intervention and control groups, which did not allow a conclusion, so future trials should ensure a clear comparison between intervention and control groups to enhance the reliability and interpretability of the findings. Finally, it should be mentioned that, based on the current state of knowledge and considering the high risk of placebo effect within mental health studies [44], further studies should include the comparison between the intervention group and the control group.

## 5. Conclusions

The evidence gathered within a systematic review of RCTs did not confirm any influence of lycopene on mental health in populations of healthy individuals and individuals with various diseases. However, only a limited number of studies have been conducted so far, so further RCTs are needed to verify the influence of lycopene provided within supplements, functional foods, and regular food products on various mental health problems in diverse populations. Moreover, further studies should include the assessment of the influence of the most effective dose to improve mental health outcomes, as well as more diverse mental health outcomes should be studied.

## Figures and Tables

**Table 1:** The population, intervention/exposure, comparator, outcome, and study design (PICOS) criteria.

PICOS Criterion	Inclusion	Exclusion
Population	Adult individuals	Pregnant or lactating women, patients with eating disorders, patients with intellectual disabilities, patients with neurological disorders
Intervention/exposure	Oral lycopene intake intervention of specified dose	Intervention including multiple factors combined
Comparison	Intervention group compared with control group	No comparison with placebo
Outcome	Any mental health result	No valid psychological measure
Study design	Study defined as Randomized Controlled Trial (RCT), published in English in peer-reviewed journal	Animal model study

**Table 2:** The detailed electronic search strategy applied to PubMed, Web of Science, Cochrane, and Google Scholar databases.

Database	The Detailed Electronic Search Strategy
PubMed	(mental health[Title/Abstract] OR mental disorder[Title/Abstract] OR mental disorders[Title/Abstract] OR psychological distress[Title/Abstract] OR mood disorders[Title/Abstract] OR depression[Title/Abstract] OR depressive[Title/Abstract] OR anxiety[Title/Abstract] OR suicide[Title/Abstract] OR suicidal[Title/Abstract] OR well-being[Title/Abstract] OR wellbeing[Title/Abstract] OR quality of life[Title/Abstract] OR self-esteem[Title/Abstract] OR self esteem[Title/Abstract] OR self-efficacy[Title/Abstract] OR self efficacy[Title/Abstract] OR resilience[Title/Abstract] OR empowerment[Title/Abstract] OR life skills[Title/Abstract] OR social participation[Title/Abstract] OR mental capital[Title/Abstract] OR emotional[Title/Abstract] OR psychosocial[Title/Abstract] OR psychology[Title/Abstract] OR psychiatry[Title/Abstract]) AND (lycopene[Title/Abstract])
Web of Science	mental health OR mental disorder OR mental disorders OR psychological distress OR mood disorders OR depression OR depressive OR anxiety OR suicide OR suicidal OR well-being OR wellbeing OR quality of life OR self-esteem OR self esteem OR self-efficacy OR self efficacy OR resilience OR empowerment OR life skills OR social participation OR mental capital OR emotional OR psychosocial OR psychology OR psychiatry (Topic) and lycopene (Topic)
Cochrane	mental health OR mental disorder OR mental disorders OR psychological distress OR mood disorders OR depression OR depressive OR anxiety OR suicide OR suicidal OR well-being OR wellbeing OR quality of life OR self-esteem OR self esteem OR self-efficacy OR self efficacy OR resilience OR empowerment OR life skills OR social participation OR mental capital OR emotional OR psychosocial OR psychology OR psychiatry in Title Abstract Keyword AND lycopene in (Title Abstract Keyword)
Google Scholar	lycopene AND mental OR psychological OR mood OR depression OR depressive OR anxiety OR suicide OR suicidal OR wellbeing OR QoL OR self-esteem OR self-efficacy OR resilience OR empowerment (Title)

**Table 3:** The extracted data to describe the Randomized Controlled Trials (RCTs) included in a systematic review.

No	Author, Year	Study Design	Country, Location	Studied Group	Time	Study Duration
[30]	Cano-Carillo et al., 2014	Double-blind, randomized, placebo-controlled clinical trial	Spain, Murcia	Patients with Burning Mouth Syndrome (BMS)	Recruitment: October 2011–May 2013	12 weeks
[31]	Morante et al., 2017	Double-blind, randomized, placebo-controlled clinical trial	Spain, Murcia	Patients with xerostomia	Not mentioned	12 weeks
[32]	Nishimura et al., 2019	Double-blind, randomized, placebo-controlled, parallel-group comparative study	Japan, Ebetsu	Healthy individuals	April–September 2018	12 weeks
[33]	Nouri et al., 2020	Double-blind, randomized, placebo-controlled clinical trial	Iran, Isfahan	Men with primary or secondary infertility	Winter and spring of 2018	12 weeks
[34]	Cormio et al., 2021	Double-blind, randomized, placebo-controlled clinical trial comparative study	Italy, Foggia *	Patients with Benign Prostatic Hyperplasia (BPH)	Recruitment 2018–2019 *	8 weeks
[35]	Quiros-Roldan et al., 2021	Double-blind, randomized, placebo-controlled clinical trial comparative study	Italy, Brescia	Human Immunodeficiency Virus (HIV)—infected patients with BPH	March–April 2020	12 weeks

* Data provided on request.

**Table 4:** The extracted data to describe the groups studied within the Randomized Controlled Trials (RCTs) included in a systematic review.

No	Number of Participants	Number of Male Participants	Number of Female Participants	Age (Intervention Group)	Age (Placebo Group)
[30]	60	12	48	61.7 ± 11.6 years	64.9 ± 14.1 years
[31]	60	7	53	64 ± 11 years	67 ± 14 years
[32]	100 (74 completed the study)	25 completed the study	49 completed the study	53.7 ± 7.8 years	53.9 ± 9.1 years
[33]	44	44	0	31.89 ± 2.51 years	32.15 ± 2.16 years
[34]	40	40	0	65.6 ± 5.1 years	64.1 ± 8.2 years
[35]	31	31	0	69 ± 8 years	63 ± 7 years

**Table 5:** The extracted data to describe the interventions applied within the Randomized Controlled Trials (RCTs) included in a systematic review.

No	Lycopene Source	Intervention	Placebo	Characteristics of Diet	Lycopene Intake (Obtained Within Intervention)
[30]	Functional food product	Lycopene-enriched extra virgin olive oil (EVOO) applied three times a day as a spray to the mouth (1.5 mL per dose), to be swallowed afterwards	Water and dye formulation applied three times a day as a spray to the mouth (1.5 mL per dose), to be swallowed afterwards	No diet characteristics	Approximately 1.35 mg per day
[31]	Functional food product	Lycopene-enriched EVOO applied three times a day as a spray to the mouth (1.5 mL per dose), to be swallowed afterwards	Water and dye formulation applied three times a day as a spray to the mouth (1.5 mL per dose), to be swallowed afterwards	No diet characteristics	Approximately 1.35 mg per day
[32]	Food product	Semidried high-lycopene tomato variety PR-7, consumed in an amount of 50 g per day	Lycopene-free tomato	Tomato consumed without cooking; previous food consumption maintained; avoiding any supplements, tomatoes, processed foods containing tomatoes, and vegetable juices (test food was the only tomato product allowed); on the basis of Food Frequency Questionnaire based on Food Groups (FFQ-G) between groups no differences in the intake of calories, proteins, lipids, carbohydrates, dietary fiber, and salt stated	22.0–27.8 mg per day
[33]	Supplement	Pills with lycopene once per day	Pills with starch	On the basis of a three-day dietary record, between groups, no differences in the intake of lycopene, fat, and protein stated	25 mg per day
[34]	Supplement	Whole tomato food supplement applied 5 g sachet daily	Orange/maltodextrin	No diet characteristics	9.5 mg per day
[35]	Supplement	Whole tomato food supplement applied 5 g sachet daily	Orange/maltodextrin	Previous food consumption maintained; avoiding any food supplements	9.5 mg per day

**Table 6:** The extracted data to describe the results of the Randomized Controlled Trials (RCTs) included in a systematic review.

No	Mental Health Outcomes	Mental Health Measures	p *	Observations (Results)
[30]	(1) Quality of life, (2) depression, (3) anxiety	(1) Short Form Health Survey (SF-36), Oral Health Impact Profile 14 (OHIP-14), (2) Hospital Anxiety and Depression (HAD), (3) HAD	(1) SF-36—*p* = 0.892, OHIP-14—*p* = 0.117, (2) *p* = 0.984, (3) *p* = 0.103	No difference between intervention and control group
[31]	Quality of life	OHIP-14	*p* = 0.84	No difference between intervention and control group
[32]	(1) Fatigue and stress, (2) mood states	(1) Visual Analog Scale (VAS) questionnaire on fatigue and stress, (2) Profile of Mood States Second Edition (POMS-2) full-length version for adults	*p* not shown, but no statistically significant difference declared	No difference between intervention and control group
[33]	(1) Depression, (2) anxiety, (3) stress, (4) quality of life	(1) Depression, Anxiety and Stress Scale—21-item questionnaire (DASS-21), (2) DASS-21, (3) DASS-21, (4) World Health Organization (WHO) Quality of Life Questionnaire (WHOQOL-BREF)	(1) *p* = 0.537, (2) *p* = 0.483, (3) *p* = 0.636, (4) psychological health—*p* = 0.640	No difference between intervention and control group
[34]	Quality of life	International Prostate Symptom Score (IPSS) questionnaire	Not mentioned	No comparison between intervention and control group
[35]	Quality of life	IPSS questionnaire	Not mentioned	No comparison between intervention and control group

* The *p*-value for the comparison between the intervention group and the control group regarding mental health measures.

**Table 7:** The assessment of the risk of bias for the Randomized Controlled Trials (RCTs) included in a systematic review, conducted based on the revised Cochrane risk-of-bias tool for randomized trials, accompanied by a summary of observations.

Ref.	D1	D2	D3	D4	D5	Overall Bias	Summary of Observations *
[30]	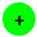	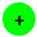	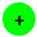	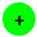	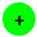	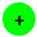	Not confirmed
[31]	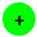	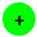	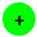	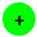	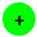	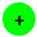	Not confirmed
[32]	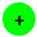	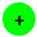	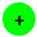	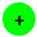	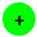	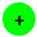	Not confirmed
[33]	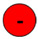	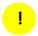	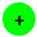	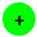	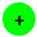	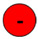	Not confirmed
[34]	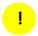	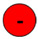	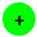	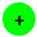	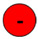	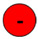	Not conclusive
[35]	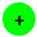	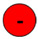	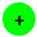	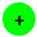	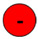	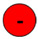	Not conclusive

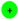
—Low risk; 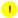
—Some concerns; 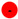
—High risk; * summary of observations defined as “Not confirmed” (if no difference between intervention group and placebo group observed), or “Not conclusive” (if no comparison between intervention group and placebo group made); D1—domain of the risk of bias arising from the randomization process; D2—domain of the risk of bias due to deviations from the intended interventions; D3—domain of the risk of bias due to missing outcome data; D4—domain of the risk of bias in measurement of the outcome; D5—domain of the risk of bias in selection of the reported result.

## Data Availability

Further inquiries can be directed to the corresponding author.

## Supplementary Materials

The following supporting information can be downloaded at: https://www.mdpi.com/article/10.3390/nu17111793/s1, Supplementary Table S1. The extracted data to describe the inclusion and exclusion criteria within the Randomized Controlled Trials (RCTs) included in a systematic review.
