# Supplementary material for: Influence of Lycopene Intake on Mental Health: A Systematic Review of Randomized Controlled Trials (RCTs)
Source: Nutrients. 2025 May 25;17(11):1793. doi: 10.3390/nu17111793 (PMC12157968; doi:10.3390/nu17111793)
Supplement: Supplementary file 1 [file nutrients-17-01793-s001.zip › nutrients-3618651-supplementary.pdf]

nutrients

Supplementary Material

Influence of Lycopene Intake on Mental Health: A Systematic Review of Randomized Controlled Trials (RCTs)

Dominika Głąbska [1], Dominika Guzek [2,*], Anna Jílková [3], Aleksandra Kołota-Burdzy [1], Dominika Skolmowska [1], and Lenka Kouřimská [3]

[1] Department of Dietetics, Institute of Human Nutrition Sciences, Warsaw University of Life Sciences (SGGW-WULS), 159C Nowoursynowska Street, 02-776 Warsaw, Poland; dominika_glabska@sggw.edu.pl (D.Gł.); aleksandra_kolota@sggw.edu.pl (A.K.B.); dominika_skolmowska@sggw.edu.pl (D.S.)
[2] Department of Food Market and Consumer Research, Institute of Human Nutrition Sciences, Warsaw University of Life Sciences (SGGW-WULS), 159C Nowoursynowska Street, 02-776 Warsaw, Poland; dominika_guzek@sggw.edu.pl (D.Gu)
[3] Department of Microbiology, Nutrition and Dietetics, Faculty of Agrobiology, Food and Natural Resources, Czech University of Life Sciences Prague, 129 Kamycka Street, 165-00 Praha, Czech Republic; jilkovaanna@af.czu.cz (A.J.); kourimska@af.czu.cz (L.K.)
* Correspondence: dominika_guzek@sggw.edu.pl; Tel.: +48-22-593-71-34

Academic Editor: Firstname Lastname

Received: date
Revised: date
Accepted: date
Published: date

Citation: To be added by editorial staff during production.

Copyright: © 2025 by the authors. Submitted for possible open access publication under the terms and conditions of the Creative Commons Attribution (CC BY) license (https://creativecommons.org/licenses/by/4.0/).

Supplementary Table S1. The extracted data to describe the inclusion and exclusion criteria within the Randomized Controlled Trials (RCTs) included to a systematic review.

No	Inclusion criteria	Exclusion criteria
[30]	A clinical history of continuous symptoms of oral burning or pain on a daily or almost daily basis, during all or part of the day for more than 6 months, without paroxysms, and independent of the nervous pathway; an absence of clinical abnormalities that might account for the symptoms; normal blood test findings (complete blood count, blood glucose, serum, iron and transferrin levels, serum vitamin B_{12}, and folate)	Pain attributable to other conditions; problems with dentures; biochemical anomalies (iron, vitamin B_{12}, folate, zinc, B complex vitamin deficiencies, and thyroid disease); history of hypersensitivity or allergy to the materials used in the study; neurological disorders, even previously treated even irregularly, with either pharmacotherapy, or psychological therapies; treatment for BMS in the last 2 weeks in the case of topical treatments or in the last 4 weeks in the case of systemic therapies
[31]	Age > 18 years; xerostomia for more than 3 months; response of 3 cm or greater on a visual analog scale (VAS) to the question about mouth being continuously dry for over 3 months; non-stimulated salivary flow rate (draining test) of less than 1.5 ml/15 min	History of hypersensitivity or allergy to the materials used in the study; history of radiation therapy to the head and neck region; diagnosis of Sjogren's syndrome; pregnancy or lactation; change of systemic medication; any hospitalization; occurrence of an uncontrolled medical condition
[32]	Age ∈ <30; 70 years); low-density lipoprotein (LDL) 0 cholesterol ∈ <120; 160 mg/dL)	Participating in the pilot study; usually do not consuming raw tomatoes; usually consuming tomato juice; being under physician's advice, treatment, and/or medication for dyslipidemia and/or diabetes; BMI ≥ 30 kg/m^2; familial hypercholesterolemia; serious cerebrovascular, cardiac, hepatic, renal, gastrointestinal diseases; infectious diseases requiring reports to the authorities; major surgical history relevant to the digestive system; unusually high or low blood pressure and/or abnormal hematological data; severe anemia; pre- or post-menopausal women complaining of obvious physical changes; risk of allergic reactions to drugs or foods; regular taking medications, functional foods, and/or supplements, which would affect blood lipid and/or glucose metabolism; alcohol addiction, eating disorder, donating either 400 mL of whole blood within 16 weeks (women) or 12 weeks (men), 200 mL of whole blood within 4 weeks (men and women), or blood components within 2 weeks (men and women) prior to the current study; pregnant or lactating women or women who expect to be pregnant during this study; participation in other clinical trial during the study or within the previous 4 weeks; any other medical and/or health reasons unfavorable to participation in the current study, as judged by the principal investigator
[33]	Infertile men; age 25–45 years; sperm count < than 20 million/mL; normal sperm <65%; volume < 3.0 mL; average motility < 60%; not receiving treatment for a period of ≥12 weeks	History of all genital diseases or disorders or genital surgery; anatomical disorders; endocrinopathy; using androgens or antiandrogens; previous hormonal therapy; using cytotoxic drugs, anticoagulants, immunosuppressants or any antioxidant supplements; physiological and psychiatric disorders that may affect sperm and sexual performance; drug abuse BMI ≥30 kg/m^2

[34]	Diagnosis of benign prostatic hyperplasia (BPH)	History of hypersensitivity to tomato; inflammatory diseases of the urogenital tract; malabsorption syndrome
[35]	Age > 18 years; HIV infection; stable antiretroviral therapy; plasmatic HIV RNA <20 copies/mL; BPH at rectal examination and trans rectal echography	Current or previous prostatic carcinoma; history of transurethral resection of the prostate; diseases of the urinary tract; inflammatory diseases of the urogenital tract; AIDS event in the previous 3 months; a history of hypersensitivity to tomato; malabsorption syndrome

[1] BMS – Patients with Burning Mouth Syndrome; BMI – body mass index; HIV – human immunodeficiency virus; AIDS – Acquired Immune Deficiency Syndrome; RNA – ribonucleic acid.

Disclaimer/Publisher's Note: The statements, opinions and data contained in all publications are solely those of the individual author(s) and contributor(s) and not of MDPI and/or the editor(s). MDPI and/or the editor(s) disclaim responsibility for any injury to people or property resulting from any ideas, methods, instructions or products referred to in the content.
